# Utility of impedance mapping to delineate atrial septal occluders during catheter ablation

**DOI:** 10.1016/j.hroo.2026.02.006

**Published:** 2026-02-12

**Authors:** Keita Yoshiyama, Yohei Kikuchi, Daiki Kumazawa, Kosuke Onodera, Yosuke Mizuno, Kennosuke Yamashita

**Affiliations:** Heart Rhythm Center, Department of Cardiovascular Medicine, Sendai Kosei Hospital, Miyagi, Japan

**Keywords:** Atrial septal occluder, Voltage mapping, Impedance mapping, Electroanatomic mapping, Catheter ablation, Atrial tachyarrhythmia, Atrial septal defect

**What We Learned From This Case**

▪ Generator-impedance mapping delineated a contiguous, stable region corresponding to the atrial septal occluder footprint on electroanatomic maps.
▪ Bipolar voltage mapping over the occluder region was heterogeneous and did not reliably define device margins.
▪ A consistent within-case impedance contrast (∼20 to 30 Ω) enabled practical demarcation of a device margin without a universal absolute threshold.
▪ Impedance-defined contours may facilitate margin-oriented ablation design for lesion placement adjacent to a definitive no-ablation surface.

## Introduction

Transcatheter closure of atrial septal defects using atrial septal occluder (ASO) devices has become the predominant therapeutic strategy in adult patients. With improved long-term survival in this population, atrial tachyarrhythmias are increasingly encountered, and catheter ablation is frequently considered as a therapeutic option. In contrast to patients with native interatrial septa, ablation in the presence of an implanted ASO is inherently constrained by a large nonmyocardial structure occupying a substantial portion of the septal anatomy. Because radiofrequency (RF) energy cannot be delivered on the device surface, ablation lesions must be intentionally placed on adjacent viable atrial myocardium while accounting for the device footprint. Under these circumstances, accurate recognition of ASO-related regions directly on electroanatomic maps (EAMs) is essential for practical ablation-line planning.^1^

In contemporary practice, anatomic understanding during ablation in patients with ASO devices relies primarily on fluoroscopy and intracardiac echocardiography (ICE). These imaging modalities provide indispensable information regarding device position, orientation, and spatial relationships with surrounding atrial structures. However, such information may not be directly incorporated into the EAM as a stable boundary, depending on the system being used, thus limiting its direct utility for catheter navigation and lesion deployment. Given that EAMs constitute the central reference framework during ablation procedures, the ability to identify device-related regions directly on these maps has important procedural implications.

Bipolar voltage mapping remains the most widely used electroanatomic parameter for substrate characterization and ablation guidance. Bipolar voltage amplitude serves as a surrogate for local myocardial activation and has proven utility for identifying scar and low-voltage regions. Nevertheless, voltage amplitude is inherently influenced by multiple factors, including wavefront direction and tissue contact.^2^ Consequently, voltage distributions over metallic occluders may appear, limiting their ability to reliably reflect anatomic boundaries. In the setting of ASO-associated ablation, these properties pose a fundamental challenge when voltage mapping is used to define a practical ablation margin adjacent to the device.

Impedance-based information provides a complementary signal domain that may overcome some of these limitations. Impedance reflects the electrical properties at the catheter–environment interface and is less dependent on activation patterns than electrogram amplitude. Previous investigations have demonstrated that impedance mapping can identify fine septal anatomy, supporting the concept that impedance reflects anatomic information not readily captured by voltage alone.^3^ From a biophysical standpoint, impedance is expected to vary systematically across blood pool, viable myocardium, and nonbiological surfaces, providing a plausible mechanism for structural discrimination during mapping.^4^ In ASO-associated ablation, where the device surface represents a definitive no-ablation zone, such information may be particularly valuable for defining device margins and guiding lesion placement on adjacent atrial tissue.

On this background, we investigated whether generator-impedance mapping obtained using an ablation catheter could provide a more stable and clinically actionable representation of ASO boundaries than bipolar voltage mapping, thereby supporting practical ablation-line planning adjacent to the device.

## Methods

We evaluated 3 patients with previous transcatheter atrial septal defect closure, who underwent catheter ablation for atrial tachyarrhythmias. Patient characteristics, arrhythmia phenotypes, and device-related information are presented in Table 1. All procedures were performed using the CARTO 3 system (Biosense Webster). High-density atrial mapping was initially performed using a multipolar mapping catheter (Octaray, Biosense Webster) to construct the chamber geometry and bipolar voltage map on the EAMs. Additional mapping focused on the device-related region was then performed using an RF ablation catheter (Thermocool SmartTouch SF catheter, Biosense Webster).

**Table 1:** Patient, arrhythmia, and device characteristics

Characteristics	Case 1	Case 2	Case 3
Age (y)	51	61	54
Gender	Male	Female	Male
Height (cm)	170	158	183
Weight (kg)	74	58.3	86.3
CHADS_2_ score	0	0	0
Arrhythmia	PeAF	PAF	AT
Number of sessions	2	1	2
Previous ablation	PVI	N/A	PVI + CTI
Energy source	Cryo	N/A	RF
Timing of ASD closure	3 y ago	12 y ago	11 y ago
ASO device	Figulla Flex II (30 mm)	Amplatzer ASO (12 mm)	Amplatzer ASO (34 mm)
Material	Nitinol mesh + ePTFE	Nitinol mesh + polyester	Nitinol mesh + polyester
LA side			
Disk area measured by CT (cm^2^)	16.4	5.55	19.8
Disk area measured by impedance map area (cm^2^)	16.8	5.7	N/A
Mean impedance on the disk (Ω)	77.0 ± 6.4	81.5 ± 4.8	N/A
Mean impedance around the rim (Ω)	101.0 ± 11.1	95.0 ± 6.2	N/A
RA side			
Disk area measured by CT (cm^2^)	12.5	3.66	14.8
Disk area measured by impedance map area (cm^2^)	14.1	4.8	14.1
Mean impedance on the disk (Ω)	83.5 ± 7.6	84.0 ± 3.2	85.0 ± 7.2
Mean impedance around the rim (Ω)	107.0 ± 8.9	88.5 ± 7.8	105.0 ± 9.5

Numbers are shown as mean ± standard deviation.

ASD = atrial septal defect; ASO = atrial septal occluder; AT = atrial tachycardia; CT = computed tomography; CTI = cavotricuspid isthmus; ePTFE = expanded polytetrafluoroethylene; LA = left atrium; N/A = not available; PAF = paroxysmal atrial fibrillation; PeAF = persistent atrial fibrillation; PVI = pulmonary vein isolation; RA = right atrium; RF = radiofrequency.

Bipolar voltage maps and generator-impedance maps were generated from the ablation catheter. Bipolar voltage maps were analyzed using standard atrial substrate mapping settings routinely applied in clinical practice (0.1–0.5 mV). Impedance mapping was based on generator impedance rather than local impedance measurements, given that the Thermocool SmartTouch SF catheter is not equipped with the microelectrodes required for local impedance assessment. Mapping points were acquired using the CARTO 3 automatic annotation algorithm, with a coronary sinus catheter used as the reference electrode. During point acquisition, contact force was maintained between 5 and 20 g to minimize contact-related variability. Within this contact force range, impedance patterns over the occluder surface remained spatially stable despite minor force fluctuations. The anatomic boundary of the ASO was visually identified using the UNIVU module (Biosense Webster) as the reference. The region defined by the impedance contrast was then verified to spatially correspond to this visual device contour (Supplemental Figure 1).

In case 3, the occluder size and limited extent of residual native septum precluded transseptal puncture away from the device. Therefore, transseptal access was achieved by puncturing through the ASO. To minimize the risk of device damage, bipolar voltage and impedance acquisition on the left atrial side were intentionally avoided.

The research reported in this article adhered to the Declaration of Helsinki, and the study was approved by the Sendai Kosei Hospital Institutional Review Board. All patients provided a written informed consent.

## Results

**Figure 1:**
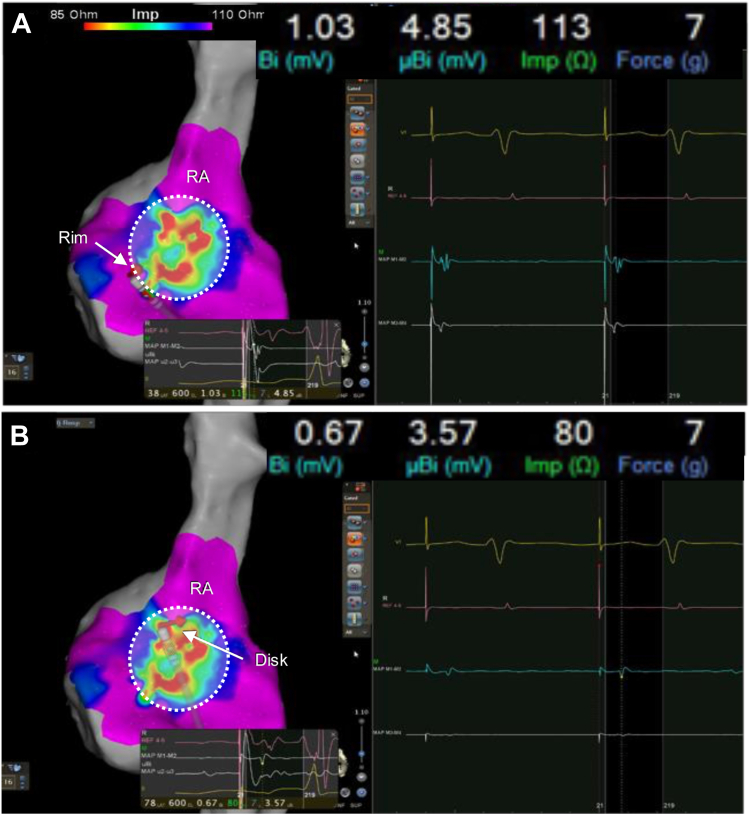
Rim and disk characteristics on the right atrial side in case 2. Representative electroanatomic data acquired on the right atrial side of the ASO in case 2. No apparent difference in bipolar voltage (1.03 and 0.67 mV) is observed between the rim (**A**) and disk (**B**) regions; however, generator impedance is lower on the disk surface (113 and 80 Ω), illustrating the ability of impedance mapping to differentiate device-related properties. Contact force was comparable between the two recordings. ASO = atrial septal occlude; Imp = impedance; RA = right atrium.

Generator-impedance mapping demonstrated a clear and reproducible difference between rim- and disk-corresponding regions (Figure 1). Across the 3 cases, generator impedance differed by approximately 20–30 Ω between the device-related region and the surrounding atrial tissue. Although absolute impedance values varied among individuals, the relative impedance gradient between device-related and adjacent atrial regions was internally consistent within each case, indicating that impedance contrast, rather than a fixed absolute cutoff underpinned boundary recognition. In contrast, bipolar voltage mapping did not provide a clinically actionable distinction between regions corresponding to the rim and the disk of the ASO. Voltage amplitudes in these regions overlapped substantially and lacked consistent spatial separation.

**Figure 2:**
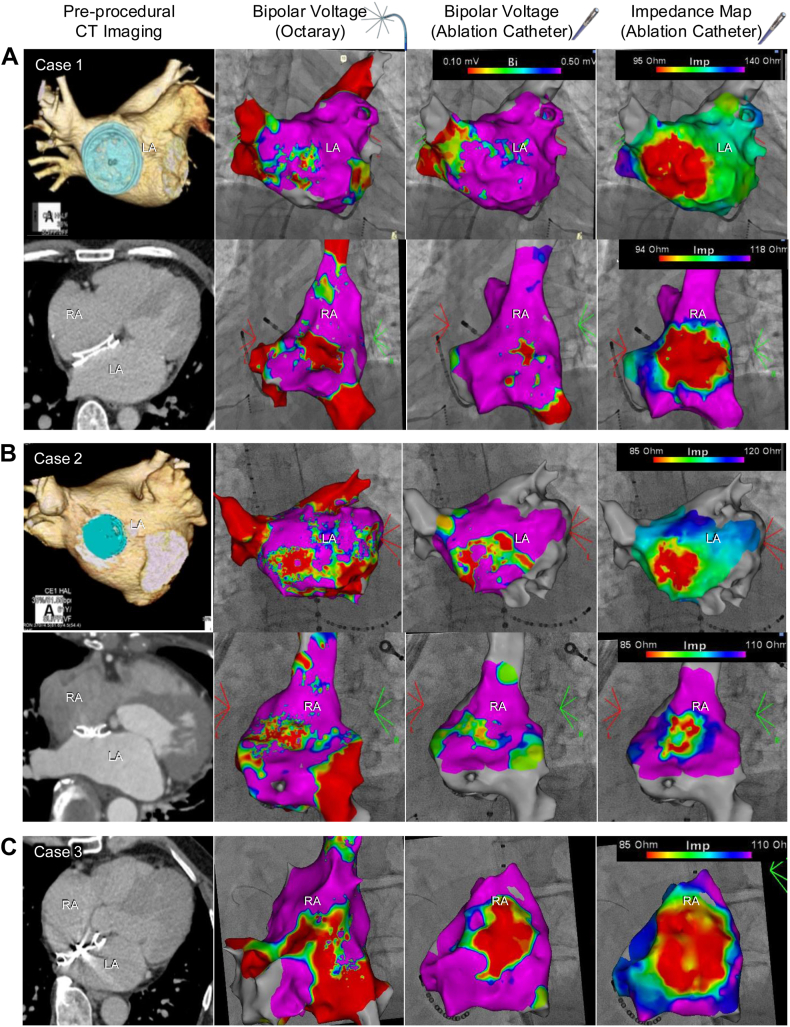
Voltage and impedance mapping of atrial septal occluder devices. Panels **A**–**C** correspond to cases 1–3. For each case, preprocedural imaging is shown in the left column, followed by representative bipolar voltage maps and a generator-impedance map acquired using the multielectrode catheter and RF ablation catheter. Bipolar voltage maps demonstrate heterogeneous and fragmented low-amplitude distributions over the occluder surface, whereas generator-impedance maps delineate a contiguous region closely matching the expected device footprint. CT = computed tomography; LA = left atrium; RA = right atrium.

Spatially, generator-impedance mapping consistently delineated a contiguous region corresponding to the expected ASO footprint on the EAMs (Figure 2). The impedance-defined region exhibited a coherent contour closely following device geometry, providing a stable representation of the device-related area. Importantly, impedance-defined contours remained stable across repeated acquisition passes. Conversely, bipolar voltage maps acquired in the same anatomic region demonstrated heterogeneous and discontinuous low-amplitude distributions. These voltage-defined regions varied with mapping density and acquisition passes, resulting in an unstable depiction of the presumed device boundary. Increasing point density and repeating mapping passes did not resolve this heterogeneity, underscoring the intrinsic limitations of voltage amplitude for representing nonmyocardial structures.

The presence of a consistent impedance difference enabled practical demarcation of a device margin on the EAMs. Because RF energy delivery cannot be performed on the occluder surface, ablation strategies must ensure that lesion sets are placed on viable atrial myocardium while maintaining separation from the device boundary. In this series, impedance-derived boundaries provided a stable spatial reference for defining such margins and facilitated the construction of ablation lines with an intentional offset from the occluder contour. Notably, this approach did not require the adoption of a universal impedance threshold; relative impedance differences within each case were sufficient to identify device-related regions and guide ablation-line planning.

## Discussion

These observations highlight a fundamental distinction between voltage- and impedance-based mapping in the setting of implanted cardiac devices. Unlike bipolar voltage, which is inherently sensitive to directional and contact-related factors as described, generator impedance reflects electrical properties at the catheter–environment interface and is less dependent on activation dynamics.^3^^,^^4^ This mechanistic difference provides a plausible explanation for the observed stability of impedance-defined boundaries over the ASO surface. Furthermore, the quantitative area of the device-related region defined by impedance mapping closely matched the disk area measured on preprocedural computed tomography (Table 1), reinforcing the anatomic accuracy of this mapping modality.

From a procedural perspective, the clinical value of impedance mapping in ASO-associated ablation lies not in achieving precise anatomic reconstruction, but in supporting practical margin delineation. In routine practice, the occluder surface constitutes a definitive no-ablation zone, and procedural planning centers on determining how close lesions can be placed to the device while remaining on viable tissue. By providing a map-integrated spatial framework on EAMs, generator-impedance mapping shifts procedural planning from voltage-based interpretation toward margin-oriented ablation design. This map-integrated margin delineation complements fluoroscopy and ICE. Although these modalities provide a critical anatomic context, manual contouring of device boundaries on the map, based on ICE, is often limited by acoustic shadowing and artifacts (Supplemental Figure 2). Therefore, ICE was used primarily for real-time visual verification rather than for creating anatomic map contours.

This approach may be particularly useful for operators performing ablation in patients with large occluder devices, limited residual native septal tissue, or complex septal anatomy where mental integration of fluoroscopic/ICE information with the EAM can be challenging.

This report is intended as a proof-of-concept and hypothesis-generating observation rather than a definitive validation study. The primary objective was not exact anatomic reconstruction, but practical margin delineation for ablation planning in the presence of a nonmyocardial device surface.

### Limitations

Several limitations should be acknowledged. This case series is small and descriptive, and clinical outcomes were not assessed. Boundary definition relied on visual contour matching using the UNIVU tool rather than predefined impedance thresholds, and interoperator variability cannot be excluded. Third, we compared generator impedance primarily with bipolar voltage mapping. As illustrated in Supplemental Video 1, unipolar voltage mapping failed to clearly delineate the device boundaries even with varying cutoffs; therefore, unipolar voltage mapping was not systematically evaluated in this study. Generator-impedance measurements may also be influenced by contact and acquisition conditions, and device surface characteristics may evolve over time, potentially affecting impedance behavior. Nevertheless, the consistent impedance patterns observed across cases suggest that generator-impedance mapping provides clinically meaningful information that complements voltage mapping. Larger prospective studies are warranted to validate these observations and to explore their applicability across different device types and mapping systems.

## Conclusion

In patients with implanted ASO devices, generator-impedance mapping provides a complementary spatial framework on EAMs, supporting practical ablation margin delineation when voltage mapping yields heterogeneous and unreliable patterns.

## Disclosures

K. Yamashita received speaker honoraria and lecture fees from Daiichi Sankyo; Johnson & Johnson/Biosense Webster; Medtronic, Japan; Abbott Medical, Japan; Japan Lifeline; and Kaneka Medix. The other authors declared no conflicts of interest.

## Footnotes

Appendix Supplementary data associated with this article can be found in the online version at https://doi.org/10.1016/j.hroo.2026.02.006.

## References

[1] Khalaph M, Lucas P, Schenker N (2025). Transseptal puncture for catheter ablation of atrial fibrillation in patients with septal occluder devices. Heart Rhythm.

[2] Josephson M.E. (2016).

[3] Pentimalli F, Cornara S, Astuti M (2021). A reliable fossa ovalis impedance mapping for safer transseptal puncture: a new vision beyond voltage. J Cardiovasc Electrophysiol.

[4] Unger LA, Schicketanz L, Oesterlein T (2022). Local electrical impedance mapping of the atria: conclusions on substrate properties and confounding factors. Front Physiol.

