# Supplementary figures and images for: Utility of impedance mapping to delineate atrial septal occluders during catheter ablation
Source: Heart Rhythm O2. 2026 Feb 12;7(4):794–8. doi: 10.1016/j.hroo.2026.02.006 (PMC13107055; doi:10.1016/j.hroo.2026.02.006)

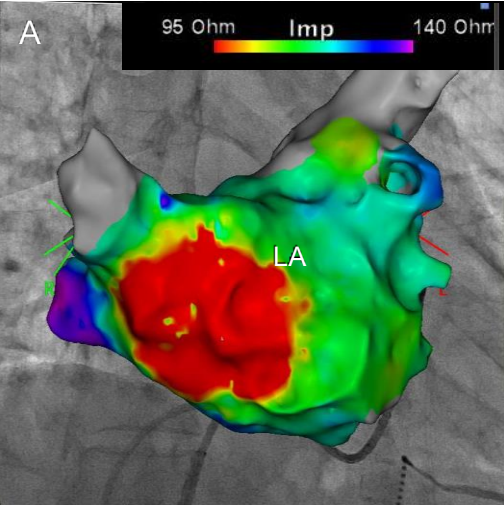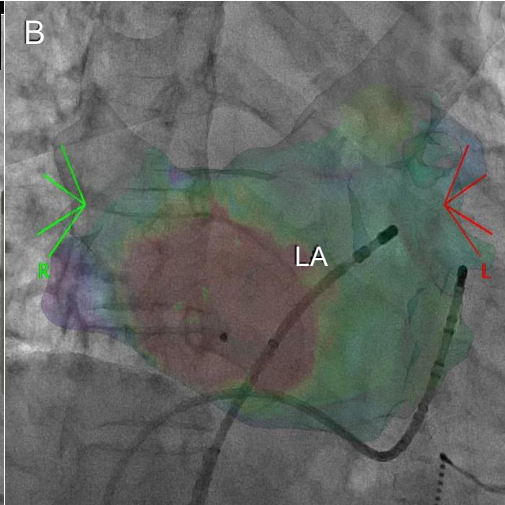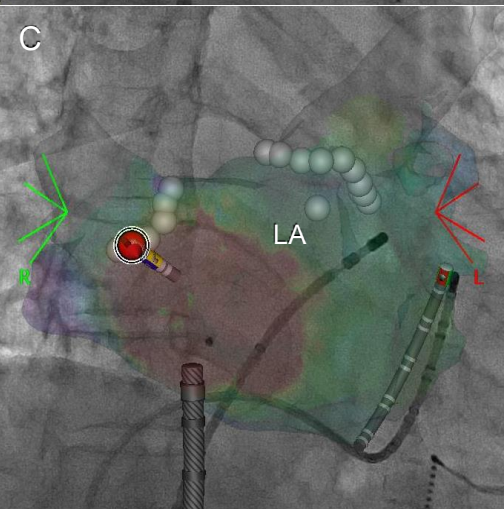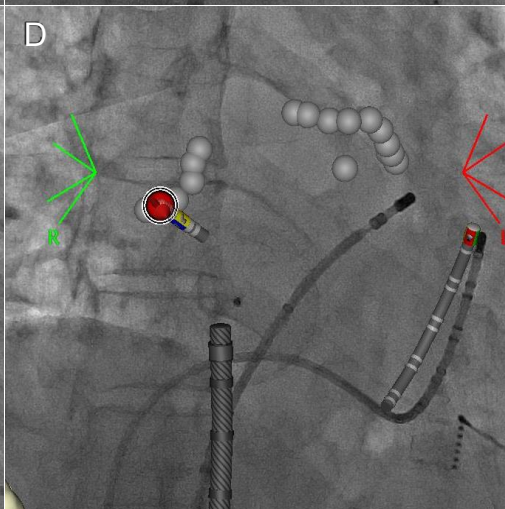

Supplement: Supplementary Figure 1 [file mmc1.pdf]

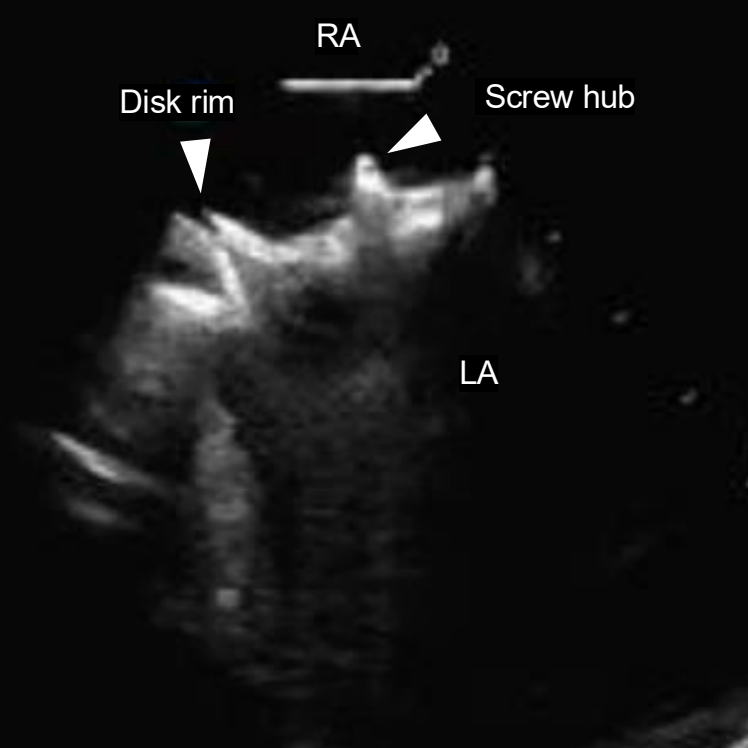

Supplement: Supplementary Figure 2 [file mmc2.pdf]
