# Supplementary material for: Utility of impedance mapping to delineate atrial septal occluders during catheter ablation
Source: Heart Rhythm O2. 2026 Feb 12;7(4):794–8. doi: 10.1016/j.hroo.2026.02.006 (PMC13107055; doi:10.1016/j.hroo.2026.02.006)
Supplement: Supplementary Material [file mmc3.docx]

**Supplementary Material Legends**

**Supplementary Figure 1. Delineation of the ASO using generator impedance mapping and UNIVU integration (Case 1).**

(A) The impedance map displayed on a three-dimensional anatomical model demonstrates delineation of the ASO geometry and rim.

(B) The same impedance map is shown with UNIVU transparency enabled and overlaid on fluoroscopy, confirming spatial correspondence between impedance-defined regions and the actual device location and shape.

(C) Ablation tags are superimposed on the impedance map, demonstrating that the isolation line was determined while maintaining a margin from the ASO rim based on impedance-derived information.

(D) Fluoroscopy with UNIVU alone, without the impedance map, shows the spatial relationship between ablation tags and the ASO; however, assessment of the distance from the ASO rim is limited with this two-dimensional visualization.

**Supplementary Figure 2. ICE imaging of the atrial septal occluder (Case 3).**

This figure presents ICE images from Case 3, demonstrating the capability and limitations of ICE for evaluation of structures surrounding the ASO. Structural components on the right atrial side, including the disk and central screw hub, can be recognized. In contrast, evaluation of the left atrial side is limited by acoustic shadowing and device-related artifacts, precluding delineation of the ASO rim and reliable assessment of distances between the device and ablation sites.

**Supplementary Video 1. Unipolar voltage mapping of the atrial septal occluder (Case 1, Right Atrium).**

This video demonstrates unipolar voltage mapping of the right atrium in Case 1. Despite varying the lower and upper voltage cutoffs across a wide range, the boundaries of the atrial septal occluder remain indistinct, and the device footprint cannot be clearly differentiated from the surrounding myocardium. This contrasts with generator-impedance mapping, which provided stable boundary visualization.
